# Extracellular Vesicles in the Gut–Vascular–Brain Axis: A Missing Mechanistic Link Between IBD and Stroke Risk

**DOI:** 10.3390/biom16040577

**Published:** 2026-04-14

**Authors:** Harshal Sawant, Erika L. Butcher, Ji Chen Bihl, Subha Arthur

**Affiliations:** Department of Biomedical Sciences, Joan C. Edwards School of Medicine, Marshall University, Huntington, WV 25755-0001, USA; sawantha@marshall.edu (H.S.); butcher111@marshall.edu (E.L.B.)

**Keywords:** inflammatory bowel disease, ischemic stroke, extracellular vesicles, exosomes, endothelial dysfunction, blood–brain barrier, systemic inflammation

## Abstract

Inflammatory bowel disease (IBD) is increasingly recognized as a systemic inflammatory disorder associated with elevated long-term risk of ischemic stroke, even among younger individuals without traditional vascular risk factors. Although chronic inflammation, endothelial dysfunction, and hypercoagulability partially explain this association, the biological mechanisms linking intestinal inflammation to cerebral vascular injury remain incompletely defined. Extracellular vesicles (EVs), membrane-bound particles released by epithelial, immune cells and platelets, have emerged as potent mediators of intercellular communication in inflammatory states. In IBD, circulating EVs are enriched with pro-inflammatory cytokines, microRNAs, adhesion molecules, tissue factors, which are capable of promoting endothelial activation, blood–brain barrier disruption, immune-thrombosis and neuroinflammation. This review summarizes epidemiologic, vascular, and EV biology literature to propose a mechanistic framework in which EV-mediated signaling integrates intestinal inflammation with cerebrovascular vulnerability along the gut–vascular–brain axis. While direct causal evidence remains limited, converging mechanistic data supports biological plausibility and defines priorities for future experimental and translational investigation.

## 1. Introduction

Epidemiological evidence consistently indicates that individuals with inflammatory bowel disease (IBD), including Crohn’s disease (CD) and ulcerative colitis (UC), experience a modest yet statistically significant elevation in stroke risk compared with the general population. A recent systematic review and meta-analysis reported that IBD is associated with an approximately 1.30-fold increased hazard of stroke, with subgroup analyses confirming elevated risk for both CD and UC [1]. Similarly, an earlier meta-analytic study reported that IBD patients have a significantly elevated risk of stroke stroke (odds ratio [OR]/relative risk [RR] ≈ 1.21), with both CD and UC contributing to increased cerebrovascular risk [2]. Large population-based cohort studies further corroborate these findings, showing that patients with biopsy-confirmed IBD have higher incident stroke rates (≈32.6 vs. 27.7 per 10,000 person-years) and an adjusted long-term hazard ratio (HR) of ~1.13 relative to matched controls, with the excess risk predominantly attributable to ischemic stroke [3]. Collectively, these evidence underscore the need to incorporate cerebrovascular risk assessment into IBD management and suggest that chronic systemic inflammation and prothrombotic pathways might contribute to the observed association.

IBD is associated with an increased risk of thromboembolic and cerebrovascular complications, including ischemic stroke, driven by chronic systemic inflammation and vascular dysfunction [4,5]. Among the emerging mechanistic contributors, extracellular vesicles (EVs), including exosomes, have gained attention as key mediators of intercellular communication in IBD. These EVs carry bioactive cargo such as proteins, lipids, and nucleic acids capable of modulating immune responses, endothelial activation, and coagulation pathways [6,7,8,9,10]. During intestinal inflammation, EVs are released in abundance and circulate widely through the bloodstream, allowing them to influence physiology far beyond their cells of origin. Their ability to transfer pathogenic cargo, including pro-inflammatory cytokines, endothelial and leukocyte adhesion molecules, and regulatory microRNAs (miRNAs), to distant vascular network positions them as plausible mediators that link intestinal inflammation to cerebrovascular injury [11,12,13].

Emerging evidence suggests that circulating EVs, such as those elevated in the systemic inflammation of IBD, contribute to thrombo-inflammatory processes and endothelial injury, central to stroke pathogenesis [12]. Notably, incidence of stroke in young or otherwise low-risk IBD patients [3], cannot be fully explained as the traditional vascular risk factors do not exist in these patients. This observation strengthens the plausibility of EVs being the compelling mechanistic link between IBD and the increased risk for stroke in the absence of vascular risk factors. Beyond their potential mechanistic role, EVs offer an integrative framework linking local intestinal inflammation with systemic vascular dysfunction, while also serving as measurable biomarkers of disease activity and presenting modifiable targets for future therapeutic intervention. Although direct clinical evidence linking IBD-derived EVs to stroke risk remains limited, growing mechanistic and experimental data supports their potential role as critical mediators connecting intestinal inflammation to cerebrovascular complications. As research continues to accelerate in this emerging topic, EVs are poised to reshape our understanding of how chronic gut inflammation contributes to cerebrovascular disease.

Although interest in IBD-associated vascular complications and the systemic roles of EVs has grown, prior reviews have largely considered these topics separately, focusing either on the epidemiologic link between IBD and stroke or on EV biology within the gut or central nervous system (CNS). As a result, the field still lacks a cohesive mechanistic model explaining how EV-mediated signaling might connect intestinal inflammation to cerebrovascular vulnerability. Given that epidemiologic data consistently shows an elevated and partially unexplained stroke risk in IBD, independent of traditional vascular risk factors, integrating these domains has become increasingly important. This review addresses that gap by proposing a unified gut-vascular-brain framework, termed the Gut-Vascular-Brain axis, in which host-derived and bacterial EVs (bEVs) transport intestine-derived inflammatory and prothrombotic cargo that may contribute to the onset of stroke among IBD patients. By reviewing emerging evidence, we aim to establish the biological plausibility of EVs as underrecognized mediators of stroke risk in IBD and to outline the mechanistic and translational research needed to determine whether EVs act as causal drivers, amplifiers, or biomarkers within this axis.

## 2. Stroke as an Emerging Extraintestinal Complication of IBD

Stroke has emerged as a clinically important extraintestinal manifestation of IBD (Table 1). Multiple epidemiologic studies, along with meta-analyses conducted over the past decade, consistently demonstrate an increased risk of both ischemic and hemorrhagic stroke among individuals with CD and UC, supporting a strong positive association between IBD and cerebrovascular events [1,5,14,15,16]. Patients with IBD have a consistently increased risk of stroke compared with the general population. Recent cohort-based meta-analyses report a significantly higher incidence of stroke in IBD, with risk estimates typically in the range of a 15–30% relative increase after adjustment for age and sex [1]. Notably, this excess risk is observed across CD (~35% increased risk of stroke) and UC (~15% increased risk of stroke), and appears to be more pronounced in younger patients, an observation that strengthens the argument for disease-specific mechanisms rather than traditional vascular risk factors alone which are less prevalent among younger population [3].

The plausibility of IBD-stroke association is reinforced by chronic immune activation characteristic of IBD, which promotes endothelial dysfunction, platelet abnormalities, and alterations in coagulation pathways, collectively contributing to a prothrombotic milieu capable of predisposing IBD patients to cerebrovascular injury [17,18,19]. Further strengthening the epidemiologic evidence, the recently published nationwide Swedish cohort study, one of the most rigorous and comprehensive investigations to date, demonstrate that more than 85,000 biopsy confirmed IBD patients experience a persistent, long-term increase in stroke risk, driven primarily by ischemic events, with a 13–14% adjusted elevation that remain significant up to 25 years after diagnosis. Notably, the increased risk is observed across CD, UC, and unclassified IBD, with the highest relative hazards detected in women and in those diagnosed at younger ages, providing robust population-level confirmation that stroke constitutes a sustained vascular complication of IBD [3].

IBD is characterized by chronic systemic inflammation, which is increasingly recognized as a driver of atherosclerosis, endothelial dysfunction, and thrombosis. In IBD, elevated circulating cytokines (e.g., Tumor necrosis factor-alpha (TNF-α), Interleukin-6 (IL-6)), C-reactive protein (CRP), and other inflammatory mediators can promote endothelial activation, oxidative stress, and plaque instability, all of which are relevant to cerebrovascular disease [20]. In parallel, IBD is associated with a hypercoagulable state, including increased platelet activation, elevated fibrinogen and factor VIII levels, reduced natural anticoagulants, and impaired fibrinolysis, which together predispose to arterial and venous thromboembolism [4]. Collectively, these inflammatory and prothrombotic pathways can affect the cerebral vessels and raise stroke risk, even without significant atherosclerosis.

Despite these well recognized associations, the mechanistic connection between intestinal inflammation and stroke remains poorly understood. Existing hypotheses, including systemic cytokines, disruption of the gut–vascular axis, and microbiome-mediated immune modulations, remain broad and largely speculative [18]. Moreover, only a subset of IBD patients develop cerebrovascular complications despite experiencing comparable inflammatory burdens, suggesting that additional factors such as genetic susceptibility, environmental exposures, or distinct immunologic endotypes may play a critical role in modulating individual risk [19]. This gap in mechanistic clarity has prompted interest in novel biological mediators capable of linking gut inflammation to distant vascular networks. Among these, exosomes, small EVs capable of transporting proteins, lipids, and nucleic acids, have emerged as a compelling but underexplored candidate [9,10]. Although direct evidence linking IBD-derived exosomes to stroke is still limited, exosomes are increasingly implicated in endothelial dysfunction, blood-brain-barrier (BBB) modulation, neuroinflammation, and thrombus formation in other cardiovascular and neurologic contexts [12,21,22,23,24,25,26]. With their ability to disseminate inflammatory signals systemically and modulate endothelial and immune cell behavior, exosomes along with other EVs may represent the missing mechanistic link in the IBD-stroke association. Before examining the potential role of IBD-derived EVs in stroke pathology, we first briefly outline the established mechanisms linking IBD to cerebrovascular risk.

## 3. Established Mechanisms Linking IBD to Cerebrovascular Risk

Building on the epidemiologic evidence linking IBD to increased stroke risk, several well-characterized biological pathways help explain how intestinal inflammation can influence cerebrovascular health. Chronic systemic inflammation is a central mechanism linking IBD to elevated cerebrovascular risk. Circulating cytokines such as TNF-α and IL-6, along with acute-phase reactants, oxidative stress, and activated immune cells, can impair endothelial nitric oxide (NO) bioavailability, destabilize atherosclerotic plaques, and accelerate vascular aging [27,28,29,30]. These inflammatory pathways contribute directly to endothelial dysfunction and atherothrombosis [28,31,32,33,34,35].

Alterations in the gut microbiome further compound this risk. IBD-associated dysbiosis leads to shifts in microbial metabolites, including trimethylamine N-oxide (TMAO), secondary bile acids, and short-chain fatty acids (SCFAs), that have been implicated in vascular inflammation, endothelial injury, and prothrombotic signaling [36,37,38,39]. These metabolic–immune interactions provide an additional route through which intestinal inflammation may influence cerebrovascular health. Beyond metabolite signaling, dysbiosis also modulates host EV biogenesis and promotes the translocation of microbial EVs into the circulation [40]. These microbiota conditioned EVs represent an additional route through which intestinal microbial shifts influence vascular inflammation and cerebrovascular susceptibility [41]. Thus, dysbiosis not only alters the cargo of host EVs but also increases systemic exposure to bEVs, creating a dual EV driven pathway along the gut–vascular–brain axis that primes the cerebral endothelium and BBB for injury [42].

Genetic and coagulation-related factors also play important roles. Patients with IBD frequently exhibit heightened platelet reactivity, elevated fibrinogen and factor VIII levels, and reduced anticoagulant activity, reflecting a systemic prothrombotic state [19,43]. Individual susceptibility likely varies based on genetic polymorphisms affecting immune regulation, endothelial responsiveness, coagulation pathways or susceptibility induced by IBD therapies [4,44]. Importantly, these modifying factors may also influence the quantity, cellular origin, and pathogenic cargo of circulating EVs in IBD. Although inflammatory burden may appear similar across patients, EV profiles may be highly heterogeneous. Differences in EV composition, such as tissue factor rich platelet EVs, epithelial derived miRNAs, or microbial antigen containing EVs, may selectively amplify thrombo-inflammatory signaling in only a subset of individuals [32,45]. Thus, the observation that only some patients develop stroke despite comparable inflammation may reflect inter-individual variability in EV biogenesis, cargo loading, or responsiveness to EV-mediated signaling, acting alongside inherited thrombophilia and vascular resilience to shape cerebrovascular vulnerability [32].

These inflammatory, microbial, and coagulation driven processes cumulatively outline a multifactorial landscape whereby IBD can heighten cerebrovascular vulnerability. This provides essential context required for the consideration of additional mechanisms that directly link and integrate IBD to stroke mechanisms such as EV-mediated signaling that may amplify these established pathways. Beyond their direct vascular effects, these metabolite shifts are expected to leave a detectable imprint on circulating EVs, including changes in EV lipid composition, a tissue factor/phosphatidylserine-rich surface phenotype, and inflammatory/Barrier-regulatory RNAs. In the following section, we outline how such metabolite-linked pathways are reflected in EV cargo during IBD and how these EVs may broadcast gut-conditioned signals to the brain vasculature.

While the systemic roles of bloodborne EVs are well established, the contribution of EVs originating specifically from the inflamed intestine remains comparatively understudied. The limited number of publications on intestine derived circulating EVs reflects an emerging research area rather than an absence of mechanistic relevance. Recent experimental data demonstrating translocation of intestinal epithelial cell derived EVs and microbial EVs across the compromised intestinal barrier during IBD support the biological plausibility that gut-origin EVs may participate in systemic vascular and neuroinflammatory processes [46,47].

## 4. EVs as Systemic Mediators and Regulators in IBD

EVs comprise a heterogeneous spectrum of membrane-bound particles, including exosomes, microvesicles, and apoptotic bodies, that differ in size, biogenesis, and functional properties [48,49]. Although these distinctions are biologically meaningful, commonly used isolation methods such as ultracentrifugation, precipitation, and size-exclusion chromatography do not reliably separate EV subtypes, resulting in mixed vesicle preparations in most studies [50,51,52]. To address this variability, the International Society for Extracellular Vesicles (ISEV) introduced the Minimal information for studies of extracellular vesicles (MISEV) guidelines, which emphasize operational terminology (e.g., “small EVs”) and standardized reporting of isolation and characterization procedures [48]. Such methodological clarity is particularly important in IBD research, where defining the specific EV populations that contribute to systemic inflammation and thrombo-inflammatory complications remains an ongoing challenge. However, because mechanistic data directly linking discrete EV subtypes to stroke pathogenesis in IBD are still limited, the present review considers EVs as a broad functional category rather than distinguishing individual vesicle classes.

Systemic inflammation in IBD, where there is an elevation of circulating EVs enriched with pro-inflammatory and pro-thrombotic cargo, is increasingly recognized as a driver of stroke pathogenesis among IBD patients. Systemic inflammatory EVs that circulate in IBD largely reflect the overall inflammatory burden of the host rather than the precise tissue source of inflammation. These EVs can originate from multiple activated cell types, such as monocytes, neutrophils, platelets, endothelial cells, and adipose tissue, and commonly carry pro-inflammatory cytokines, chemokines, and inflammation-associated miRNAs [53]. For example, circulating EVs enriched in TNF-α, IL-6, or miR-146a have been detected in IBD patients and correlate with disease activity, but similar signatures are also observed in other systemic inflammatory conditions, including rheumatoid arthritis, acute lung injury and sepsis [54,55]. As such, while these systemic EVs serve as useful biomarkers of generalized immune activation, they provide limited insight into gut-specific pathogenic processes [56].

In contrast, intestinal-origin EVs convey molecular information uniquely produced within the inflamed gut and thus more directly reflect local intestinal pathology. Intestinal epithelial cells (IECs), immune cells residing in the lamina propria, and gut microbiota all release EVs that may enter the systemic circulation, particularly when epithelial barrier integrity is compromised. For instance, EVs derived from inflamed IECs are known to be enriched in epithelial-specific miRNAs such as miR-21 and miR-155, both of which are upregulated in active IBD [57,58]. miR-21 containing EVs can amplify inflammatory signaling by suppressing anti-inflammatory targets in recipient immune or endothelial cells, while miR-155 has been shown to promote macrophage activation and endothelial dysfunction [59,60,61]. The presence of such miRNAs within circulating EVs suggests a direct molecular mechanism through which intestinal inflammation can influence distant organs.

Beyond miRNAs, intestinal EVs also carry danger-associated molecular patterns (DAMPs) released by stressed or damaged epithelial cells. These include molecules such as High-mobility group box 1 (HMGB1) and heat-shock proteins, which can activate Toll-like receptors (TLRs) and pattern recognition receptors on vascular endothelial cells [62]. When delivered systemically via EVs, these DAMPs may promote endothelial activation, oxidative stress, and pro-thrombotic states-processes that are central to cerebrovascular injury.

More recently, long non-coding RNAs (lncRNAs) packaged into intestinal EVs have emerged as additional pathogenic mediators. Certain lncRNAs released from inflamed intestinal tissue have been shown to promote the formation of neutrophil extracellular traps (NETs) by priming neutrophils toward a hyper-responsive state [25]. NET formation has been implicated in both intestinal tissue damage and cerebral microvascular thrombosis; thus, EV-mediated transfer of pro-NETotic lncRNAs provides a plausible mechanistic link between gut inflammation and downstream neurovascular complications.

Importantly, the inflamed gut also becomes a source of bEVs. During active IBD, increased intestinal permeability allows bEVs containing lipopolysaccharides, (LPS), peptidoglycans, and other microbial antigens to translocate into the circulation [63,64]. These bEVs can cross the BBB or activate cerebral endothelial cells indirectly through systemic immune activation. For example, bEV-associated LPS has been shown to induce endothelial inflammation and coagulation pathway activation, processes that are highly relevant to ischemic stroke and other forms of cerebrovascular injury. In addition, dysbiosis associated metabolites such as TMAO have been shown to enhance the release of tissue factor rich and phosphatidylserine positive EVs from endothelial and immune cells, thereby increasing their thrombo-inflammatory potential [65]. Reduced SCFA availability, conversely, diminishes anti-inflammatory EV miRNA cargo and alters EV lipid profiles toward a more pro-thrombotic phenotype [66]. Together, these findings indicate that the altered intestinal metabolic and microbial environment in IBD is reflected in EV cargo composition, suggesting that circulating EVs carry gut-conditioned signals capable of influencing distant vascular beds, including the cerebral microvasculature [64].

Collectively, these observations underscore a critical distinction that while systemic inflammatory EVs reflect a non-specific inflammatory state, intestinal-origin EVs provide gut-specific molecular signatures that mirror epithelial damage, microbial dysbiosis, and immune dysregulation within the intestine. By transporting epithelial miRNAs, DAMPs, pro-NETotic lncRNAs, and microbial antigens from the gut to the circulation, these EVs establish a direct mechanistic pathway linking intestinal inflammation to cerebrovascular dysfunction. Consequently, intestinal-derived EVs may represent not only more informative biomarkers of disease origin but also actionable mediators in the gut-brain-vascular axis in IBD.

A growing body of evidence shows that during active IBD, EVs are significantly elevated not only in serum, but also in saliva and feces [67,68]. Individuals with CD or UC exhibit higher plasma concentrations of EVs carrying cytokines (e.g., IL-6, IL-1β, TNF-α), miRNAs linked to endothelial dysfunction and coagulation (e.g., miR-155, miR-21, and miR-223), and adhesion molecules including intercellular Adhesion Molecule 1 (ICAM-1) and E-selectin [68,69,70]. Interestingly, circulating EV profiles differ markedly in patients during IBD remission. In remission, EV levels decrease and their inflammatory cargo is reduced, though not always normalized, indicating persistent low-grade immune activation [10,67]. These dynamic shifts in EV abundance and molecular cargo closely mirror fluctuations in disease activity and may play a role in extraintestinal complications, particularly endothelial injury and thrombosis.

Although direct experimental evidence implicating IBD-derived EVs as initiators of stroke pathology is still lacking, these findings however suggest that EVs are not merely passive biomarkers of IBD mediated systemic inflammation but are potential active contributors to the pathological crosstalk between chronic intestinal inflammation and the vasculature. By serving as molecular messengers that disseminate inflammatory and pro-thrombotic signals beyond the gut, EVs may help explain why individuals with IBD face an elevated risk of stroke, positioning them as both indicators and potential mediators of this heightened cerebrovascular susceptibility.

## 5. Cellular Sources of Pathogenic EVs in IBD

EVs released during intestinal inflammation differ markedly according to their cellular origin and molecular cargo. In IBD, epithelial cells, immune cells, platelets, and the gut microbiome each contribute distinct EV populations that encode signals reflecting mucosal injury, immune activation, dysbiosis, and coagulation imbalance. Rather than acting independently, these vesicles represent complementary layers of gut-derived information conveyed into the systemic circulation [7,46].

### 5.1. EVs Derived from Intestinal Epithelial Cells

As the first line of contact with luminal stressors, IECs are uniquely positioned to shape both local and systemic EV signaling in IBD. IECs are a prominent and well-characterized source of circulating EVs during IBD. Under the influence of mucosal inflammatory cytokines and cellular stress, IECs release EVs enriched with DAMPs and pro-inflammatory miRNAs such as miR-21 and miR-155 that are heavily implicated in endothelial activation and vascular injury in inflammatory settings [8,71]. IEC-derived EVs carry molecules that are implicated in adhesion and antigen presentation, such as major histocompatibility complex (MHC) class I molecules, MHC class II molecules, CD63, CD26/dipeptidyl peptidase IV. These support adhesion and antigen presentation, thereby amplifying systemic immune activation that can secondarily contribute to vascular inflammation [11,72]. Moreover, during disease flares, increased intestinal permeability likely facilitates the escape of epithelial-derived EVs into the systemic circulation, where they can interact with endothelial cells, promoting activation, cytokine release, and vascular inflammation [9]. As mentioned in Section 4, EVs containing LncRNA LINC00668 in the inflamed intestine are in fact secreted by IEC. This emerging pathway directly links epithelial-derived EVs to NET-mediated thrombosis, providing a mechanistic bridge between mucosal inflammation and the heightened thrombotic risk observed in IBD [25]. This NET-mediated immune-thrombosis is a central contributor to ischemic cerebrovascular injury, suggesting that gut-derived EVs may not only reflect intestinal inflammation but actively participate in establishing a pro-thrombotic, stroke-prone vascular environment in IBD [17,73,74,75].

While epithelial EVs initiate and broadcast mucosal danger signals, immune cell-derived EVs intensify and propagate these signals within the inflamed microenvironment and into the systemic circulation [76].

### 5.2. EVs Derived from Immune Cells

Immune cells are another major source of EVs during intestinal inflammation, and this becomes particularly evident in IBD. Several reviews on EVs in IBD highlight that, during flares, immune cells in the gut mucosa markedly increase exosome release [6,8,9,10,11]. These EVs are enriched with pro-inflammatory cytokines, oxidative enzymes, DAMPs, and pro-coagulant lipids and proteins, allowing them to function as systemic mediators of thrombo-inflammation rather than merely local signaling particles [77,78,79,80]. Experimental studies show that inflammatory EVs activate endothelial cells and enhance adhesion molecule expression, promoting leukocyte–endothelial interactions and vascular inflammation [81,82]. Persistent exposure to this EV-driven thrombo-inflammatory environment can sensitize the vasculature to arterial thrombosis, including within the cerebral circulation [83,84].

Macrophage- and monocyte-derived EVs are particularly abundant during active IBD and are enriched with pro-inflammatory cytokines and miRNAs that can potentially regulate endothelial activation and inflammatory signaling cascades [85,86,87,88,89,90]. Inflammatory EVs can induce upregulation of vascular adhesion molecules and enhance leukocyte-endothelial interactions, thereby amplifying systemic vascular inflammation [91,92,93]. Dendritic cell-derived EVs, which are increased in inflamed intestinal tissue, carry MHC molecules and immunostimulatory proteins capable of sustaining immune activation beyond the gut [94,95]. Such persistent immune signaling may contribute to endothelial stress and thrombo-inflammatory priming relevant to cerebrovascular injury.

The pro-inflammatory environment shaped by epithelial and immune EVs effectively primes platelet activation, after which platelet-derived EVs emerge as major downstream drivers of coagulation and immune-thrombotic processes.

### 5.3. Platelet Derived EVs

Platelet-derived EVs represent a critical mechanistic bridge between intestinal inflammation and thrombosis. IBD is characterized by persistent platelet activation and a prothrombotic shift in hemostasis, including elevated fibrinogen and factor VIII, reduced natural anticoagulants, and heightened platelet-leukocyte interactions, features that favor vesiculation and systemic dissemination of platelet-derived EVs [17,19]. These platelet EVs are enriched in phosphatidylserine and tissue factor, providing catalytic surfaces that accelerate thrombin generation and fibrin formation [96,97]. In parallel, platelet EVs express adhesion determinants (e.g., P-selectin) that support platelet-leukocyte aggregates and endothelial adhesion, further amplifying vascular inflammation and coagulation. In the inflammatory milieu of IBD, sustained platelet activation therefore chronically elevates the circulating burden of procoagulant platelet EVs, lowering the threshold for thrombus initiation in systemic and cerebral vascular beds.

Importantly, platelet EVs have been directly implicated in arterial thrombosis and ischemic stroke pathophysiology in non-IBD populations, underscoring their cerebrovascular relevance and supporting biological plausibility within the gut-vascular-brain axis [98,99,100,101,102,103,104,105,106,107]. In IBD, platelet EV-driven coagulation acceleration acts synergistically with (i) endothelial priming by inflammatory host EV cargo (e.g., miR-155/miR-21; cytokines) and (ii) innate immune activation triggered by bEVs (e.g., TLR2/4, cyclic GMP-AMP synthase- stimulator of interferon genes (cGAS–STING)), culminating in a pro-thrombotic, immunoinflammatory vascular state that predisposes to ischemic stroke. This triangulation of host EVs, bEVs, and platelet EVs is consistent with epidemiology showing a disproportionate increase in ischemic (over hemorrhagic) stroke risk in IBD, particularly among younger patients with fewer traditional atherosclerotic risk factors, suggesting that thrombo-inflammatory vesicle biology rather than plaque burden may be the dominant driver of cerebrovascular vulnerability in this setting.

### 5.4. Gut Microbiome Derived EVs (bEVs)

Beyond host derived EVs, bacterial EVs are considered as integral components of the gut-vascular-brain axis because they can (i) traverse a compromised intestinal barrier, (ii) transport microbial ligands (e.g., LPS, lipoproteins, peptidoglycans, nucleic acids), and (iii) engage endothelial, immune, and neurovascular receptors to amplify thrombo-inflammation and BBB vulnerability [41,108]. Emerging evidence indicates that gut microbiota profoundly shapes EV biogenesis and cargo composition during intestinal inflammation, and these microbiome-conditioned EVs may act as key mediators in gut-vascular-brain communication [109]. Moreover, dysbiosis in IBD alters microbial metabolites such as TMAO, SCFAs, and bile acid derivatives, which in turn regulate host EV cargo by modulating epithelial and immune cell metabolic and inflammatory pathways. For example, TMAO enhances endothelial inflammatory signaling and promotes tissue factor-rich EV release, while reduced SCFA levels diminish anti-inflammatory EV miRNA content and impair barrier-protective signaling [65,66]. Additionally, the bEVs that enter systemic circulation carrying microbial lipoproteins, LPS, peptidoglycans, and microbial nucleic acids, can activate TLR2, TLR4, NOD-, LRR- and pyrin domain-containing protein 3 (NLRP3), and cGAS–STING pathways in endothelial cells and microglia, thereby amplifying neuroinflammatory responses [39,110]. Experimental studies show that bEVs from gut pathogens exacerbate colitis and endothelial injury, whereas bEVs from beneficial species modulate tryptophan metabolism, reduce microglial activation, and improve cognitive outcomes [111,112,113].

Collectively, microbiota modulated EV cargo, including pro-inflammatory miRNAs, DAMPs, microbial antigens, and thrombogenic lipids, provides a mechanistic conduit through which dysbiosis in IBD may intensify endothelial dysfunction, promote BBB breakdown, and amplify neuroinflammatory pathways relevant to stroke pathogenesis.

### 5.5. Stroke Relevant EV Cargo in IBD

While Section 5.1, Section 5.2, Section 5.3 and Section 5.4 focus on the cellular sources of EVs, the pathogenic relevance to stroke ultimately depends on the molecular cargo they carry. Across EV populations in IBD, convergent cargo signatures likely shape cerebrovascular vulnerability by coordinating endothelial priming, coagulation, leukocyte adhesion, and neuroinflammation. Prioritizable candidates include the following: miRNAs such as miR-155 and miR-21 that are produced in IBD intestine and implicated in stroke pathogenesis [85,114,115]; increased tissue factor and phosphatidylserine in IBD, which accelerate thrombin generation and fibrin formation as seen in the pathogenesis of stroke [17,116]; P-selectin, significantly higher during both the active and inactive phases in patients with IBD, promotes platelet- leukocyte aggregates and immune-thrombosis that drives acute systemic stroke [97,99,105,117]; DAMPs such as calprotectin that is released during tissue damage in IBD, activate TLR/receptor for advanced glycation end products (RAGE) pathways in endothelium and microglia driving stroke onset [62,118]; intestinal cells derived cytokines sustain endothelial priming and neuroinflammatory amplification [4]; oxidized phospholipids and sphingolipids produced during IBD can impair the barrier integrity in endothelial cells [119,120]; and lncRNAs that trigger NETosis lower the threshold for occlusive immune-thrombosis in cerebral microvessels [25]. These discrete cargo pathway linkages provide a mechanistic bridge from intestinal inflammation to stroke relevant vascular phenotypes and encourage EV based molecular profiling to identify actionable signatures for risk stratification and intervention in IBD.

Thus, integrating the microbiome–EV axis into existing models of gut-vascular-brain communication offers a more complete mechanistic framework explaining how dysbiosis in IBD may potentiate neurovascular injury and stroke risk.

## 6. EV Mediated Mechanisms Linking IBD to Cerebrovascular Injury

**Figure 1:**
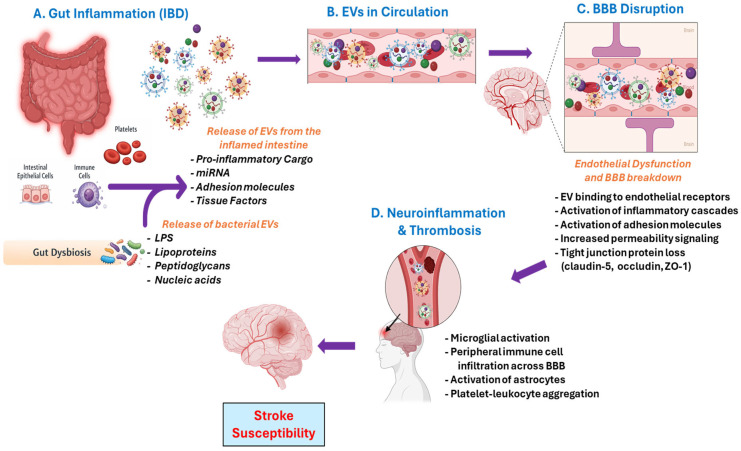
Extracellular Vesicle-Mediated Gut–Vascular–Brain Axis in IBD-Associated Stroke Risk. (**A**) IBD leads to chronic gut inflammation driving the release of extracellular vesicles (EVs) enriched with pro-inflammatory cargo, including cytokines, miRNAs, adhesion molecules, and tissue factors from the cellular sources of the intestine. IBD related dysbiosis also results in the release of bEVs carrying lipoproteins, LPS, peptidoglycans, and microbial nucleic acids. (**B**) These EVs enter systemic circulation, where they interact with vascular and immune cells. (**C**) Circulating EVs reach the cerebral vasculature and contribute to BBB disruption through endothelial receptor binding, increased permeability signaling, and loss of tight junction proteins (claudin-5, occludin, ZO-1). (**D**) BBB breakdown promotes neuroinflammation and thrombosis, characterized by microglial activation, astrocyte reactivity, peripheral immune cell infiltration, and platelet-leukocyte aggregation. Together, these processes increase overall stroke susceptibility in the context of IBD.

Although direct experimental evidence linking IBD derived EVs to stroke is still limited, a growing body of work in vascular and neuroinflammatory models shows that EVs can profoundly influence endothelial function, BBB integrity, neuroimmune activation, and thrombo-inflammatory signaling, processes that are highly relevant to stroke biology (Figure 1). These converging findings provide a rationale for examining how EV-mediated signaling may connect intestinal inflammation with cerebrovascular vulnerability in IBD.

### 6.1. EV Mediated Endothelial Dysfunction

Endothelial dysfunction represents a critical initiative step in ischemic stroke pathogenesis, and EVs have emerged as potent modulators of this process [121,122,123]. Inflammatory EVs can directly activate endothelial cells by delivering cytokines, miRNAs, and other bioactive molecules that trigger inflammatory signaling cascades [56,124,125,126]. Experimental studies demonstrate that such EVs induce the expression of adhesion molecules including ICAM-1 and vascular cell adhesion molecule-1 (VCAM-1), activate nuclear factor kappa B (NF-κB) dependent transcriptional programs, and impair endothelial NO synthase (eNOS) activity, collectively shifting the endothelium toward a pro-adhesive, vasoconstrictive, and pro-thrombotic state [127,128,129,130]. These changes are particularly consequential in the cerebral microvasculature, where endothelial cells maintain the BBB and regulate cerebral blood flow. In addition, disruption of NO homeostasis, increased leukocyte-endothelial interactions and heightened inflammatory signaling weaken vascular resilience and lower the threshold for ischemic injury [127,131,132,133,134,135].

Systemic inflammatory conditions can further amplify this vulnerability through release of circulating EVs enriched with TNF-α responsive miRNAs have been shown to compromise endothelial barrier integrity, downregulate tight junction proteins, and enhance leukocyte adhesion [125]. In the context of IBD, where surges in pro-inflammatory cytokines and circulating EVs accompany disease flares, similar vesicle-mediated endothelial priming is likely to occur within the cerebral circulation in IBD patients [136]. This persistent inflammatory pressure would sensitize cerebral endothelium to subsequent insults, promoting microvascular dysfunction even in the absence of traditional atherosclerotic disease. Together, these mechanisms support a biologically plausible hypothesis that helps explain the elevated ischemic stroke risk observed in IBD, by positioning EV mediated endothelial dysregulation as a putative mediator connecting intestinal inflammation to cerebrovascular vulnerability.

### 6.2. Blood–Brain Barrier Vulnerability

The BBB plays a critical role in maintaining cerebral homeostasis and limiting neuroinflammation. Emerging evidence demonstrates that EVs can modulate BBB integrity by altering tight junction protein expression, including occludin, claudin-5, and zonula occludens-1 (ZO-1) [137]. In experimental stroke models, pro-inflammatory EVs exacerbate BBB permeability and worsen infarct volume [45,138,139]. This disruption allows for greater leukocyte infiltration and the influx of serum proteins into the brain parenchyma, resulting in further damage. A previous study of the UC disease model showed that *Akkermansia muciniphila* derived EVs impaired intestinal and BBB integrity by upregulating tight junction proteins, suppressed neuroinflammation via reduced hippocampal pro-inflammatory cytokines, and inhibited microglial/astrocyte activation [111]. MiRNAs (e.g., miR-155) enriched in intracellular vesicles have been implicated in tight junction disruption and endothelial cytoskeletal remodeling [140]. Moreover, microbial EVs enriched in LPS, lipoproteins, and peptidoglycans have been shown to disrupt tight junction integrity via TLR4 and myeloid differentiation primary response 88 (MyD88) dependent pathways, suggesting a plausible mechanism by which dysbiosis-driven vesicles may exacerbate BBB fragility in IBD. Although direct causal data in IBD are limited, experimental EV studies demonstrate tight-junction modulation and barrier weakening (e.g., altered occludin/claudin-5/ZO-1) in vascular and neurovascular models [45,111,138,139,141]. Accordingly, we propose a mechanistic hypothesis whereby chronic exposure to circulating inflammatory EVs, together with microbiome-derived bEVs that activate TLR/cGAS–STING pathways, primes the BBB for injury in IBD, such that subsequent vascular occlusion yields greater permeability and neuroinflammatory amplification.

### 6.3. Neuroinflammation

Beyond vascular effects, EVs influence neuroinflammatory pathways. EVs have been shown to activate microglia and astrocytes, promoting the release of IL-1β, TNF-α, and reactive oxygen species (ROS) that exacerbate neuronal injury after ischemia [45,141]. Moreover, dysbiosis derived EVs can activate microglia through TLR and cGAS–STING signaling, amplifying neuroinflammation in a manner highly relevant to stroke outcomes [39,110]. Additionally, microglial priming is increasingly recognized as a determinant of neuroinflammation [102]. Likewise, the systemic inflammatory condition induced by IBD may heighten cerebral vulnerability prior to ischemia. Given the systemic inflammatory burden in IBD and the documented capacity of EVs to cross or signal across the BBB, EV-mediated neuroimmune activation represents a plausible contributor to heightened cerebrovascular susceptibility in IBD.

### 6.4. Pro-Thrombotic EV Signaling and Ischemic Stroke Initiation

Circulating EVs enriched in tissue factors and phosphatidylserine, which act synergistically to initiate and propagate blood coagulation, accelerate thrombin generation and fibrin formation in both venous and arterial thrombosis models [101,103,104,105,106,107]. In IBD, platelet activation and immune cell–derived vesicle release contribute to a procoagulant systemic environment [142]. Additionally, as mentioned earlier, recent experimental work demonstrates that intestinal IEC derived EVs can promote NET formation, linking mucosal inflammation to immunothrombosis [25]. NETs are increasingly recognized as central contributors to ischemic stroke pathogenesis through platelet activation and fibrin stabilization [141].

Taken together, these converging lines of evidence underscore the plausibility that EV-mediated signaling serves as a mechanistic bridge linking intestinal inflammation to heightened cerebrovascular vulnerability in IBD (Table 2). Although direct experimental proof is still needed, the cumulative data from endothelial, BBB, neuroimmune, and thrombo-inflammatory studies point toward a unifying model in which chronic exposure to pro-inflammatory EVs primes the cerebral microenvironment for injury long before an ischemic event occurs. By shaping vascular tone, barrier integrity, glial reactivity, and coagulation dynamics, EVs may orchestrate a multi-level disruption along the gut–vascular–brain axis that ultimately lowers the threshold for stroke initiation and worsens its consequences. Elucidating these pathways will be essential not only for clarifying the biological basis of stroke risk in IBD but also for identifying novel biomarkers and therapeutic targets capable of interrupting EV-driven cerebrovascular injury.

## 7. Are EVs Drivers, Amplifiers or Biomarkers of Stroke Risk in IBD?

A central unresolved question is whether EVs function as causal drivers of cerebrovascular injury in IBD or as secondary amplifiers of systemic inflammation, or merely as biomarkers reflecting inflammatory burden. Clarifying this distinction determines whether EVs represent therapeutic targets, mechanistic intermediates, or indicators of risk.

Biological plausibility for a causal role is strong. As described in Section 6, EVs are actively secreted during immune and epithelial activation, carry defined molecular cargo capable of reprogramming recipient cells, and have established pathogenic roles in thrombo-inflammatory conditions such as atherosclerosis, sepsis, and acute ischemic stroke. In IBD, circulating EVs are enriched with tissue factors, adhesion molecules, and inflammatory miRNAs that could directly modulate endothelial NF-κB signaling, NO bioavailability, and coagulation cascades, pathways central to ischemic stroke pathogenesis [10,46,68]. Experimental vascular models demonstrate that pro-inflammatory EVs impair endothelial barrier integrity and enhance thrombin generation, providing mechanistic coherence between EV biology and cerebrovascular vulnerability.

Despite these compelling links, direct causal evidence in IBD-associated stroke remains limited. So far, no prospective studies have demonstrated that EV burden or cargo alterations precede cerebrovascular events in IBD cohorts. Dose-response relationships remain undefined, and interventional evidence showing that EV modulation alters stroke outcomes is lacking. In addition, EV heterogeneity complicates interpretation; pathogenic subsets may coexist with EVs that exert homeostatic or anti-inflammatory functions. Without mechanistic discrimination, aggregate EV measurements can blur signal and noise.

An alternative and perhaps more immediately probable model is that EVs function as systemic amplifiers rather than primary initiators. Chronic intestinal inflammation may generate a circulating EV milieu that “primes” endothelial cells, platelets, and innate immune pathways, lowering the threshold for thrombus formation or BBB disruption in the presence of secondary vascular stressors. This would indicate that EVs do not independently cause stroke but reshape vascular susceptibility. Such priming could explain why cerebrovascular risk is disproportionately elevated in younger IBD patients lacking conventional atherosclerotic burden.

A third possibility is that EV signatures function predominantly as high-resolution biomarkers of systemic inflammation rather than direct mediators of vascular injury. Distinguishing these roles will require mechanistically grounded studies. Key priorities include longitudinal EV profiling with adjudicated cerebrovascular outcomes, integrated IBD–stroke models to test two-hit vulnerability where gut inflammation primes the system before a cerebrovascular insult, and cargo-specific gain- and loss-of-function in-vitro experiments to determine which EV constituents are sufficient or necessary to drive pathogenic phenotypes.

To bridge the other evidence gaps, direct experimental validation is needed. Controlled EV transfer studies using EVs isolated from inflamed intestinal tissue or from IBD patient plasma and administering them to naive animals or in-vitro cerebrovascular models would allow testing of EV sufficiency for endothelial activation, BBB disruption, and thrombo-inflammatory responses [46]. Similarly, dual-hit (IBD-stroke) mouse models, in which intestinal inflammation precedes cerebral ischemia, could reveal whether IBD-associated EVs exacerbate infarct severity or impair cerebrovascular resilience [4]. Complementary EV-inhibition approaches, such as pharmacologic suppression of EV biogenesis (e.g., neutral sphingomyelinase 2 (nSMase2) inhibitors) or antibody mediated neutralization of pro-thrombotic EV cargo, would help determine whether blocking EV signaling attenuates stroke-relevant vascular injury [147,148]. Together, these experimental strategies are essential for moving the field from correlative associations toward determining whether EVs act as drivers, amplifiers, or biomarkers within the IBD-stroke axis.

## 8. Research Priorities for Defining EV-Mediated Stroke Risk in IBD 

The central challenge facing this field is not whether EVs are elevated in IBD, but whether EV-mediated signaling constitutes a mechanistically necessary link between intestinal inflammation and cerebrovascular injury. Addressing this question requires a decisive shift from descriptive profiling toward causal interrogation.

The first strategic priority is to establish temporality and predictive validity. Temporality requires that EV alterations occur before cerebrovascular events, demonstrating that vesicle changes are not merely consequences of acute injury. Predictive validity requires that these EV signatures forecast future stroke risk and add information beyond established vascular and inflammatory risk factors. Large, longitudinal IBD cohorts with adjudicated cerebrovascular outcomes must therefore incorporate standardized EV profiling at serial time points [149,150]. Demonstrating that specific EV signatures precede ischemic events and independently predict stroke beyond traditional vascular risk factors would provide critical evidence that vesicle biology is not merely reflective of inflammatory burden [45]. This will further facilitate the integration of EV metrics into existing biobank infrastructures and therapeutic registries, a feasible path forward toward patient specific treatment strategies [151].

Second, the field requires mechanistic sufficiency testing in dual-hit experimental systems. Combined IBD-stroke animal models should be developed to determine whether IBD-derived EVs exacerbate cerebral ischemic injury, alter infarct size, or worsen BBB disruption. Gain- and loss-of-function approaches with defined vesicle cargo will be essential to establish pathology [152,153]. Without experimental demonstration that EV manipulation alters cerebrovascular outcomes, causal inference will remain incomplete.

Third, technological advancement must be leveraged to resolve vesicle heterogeneity. Bulk EV quantification obscures biologically meaningful subpopulations and risks combining distinct functional phenotypes into a single aggregate signal. Single-vesicle multi-omics, high-resolution flow cytometry, and spatial transcriptomic approaches make it possible to identify pathogenic EV subsets with cell-of-origin specificity and discrete molecular signatures [154,155]. Discriminating between inflammatory, thrombotic, and potentially protective vesicle populations will be prerequisite for translational targeting and for determining which EV subsets truly contribute to cerebrovascular vulnerability.

Fourth, the gut-vascular-brain axis should be conceptualized within a system’s biology framework. EV signaling does not operate in isolation. It intersects microbiome-derived metabolites, coagulation cascades, endothelial metabolic reprogramming, and neuroimmune activation. Mapping these interconnected pathways with integrative network modeling may reveal convergent nodes of vulnerability that are more tractable therapeutic targets than EV suppression alone [156,157].

Finally, what constitutes clinical relevance has to be defined. The goal is not simply to catalog EVs, but to determine whether EV biology can refine risk stratification, guide preventive strategies, or reveal modifiable determinants of cerebrovascular injury. Translational success will require demonstration that EV based metrics provide incremental value beyond established inflammatory and coagulation biomarkers, i.e., they must predict risk or inform therapeutic management more effectively than the tools clinicians already use [42,158,159,160]. It will also require showing that EV profiling meaningfully improves clinical decision-making rather than adding descriptive complexity, offering actionable insights that change how patients are monitored or treated.

**Figure 2:**
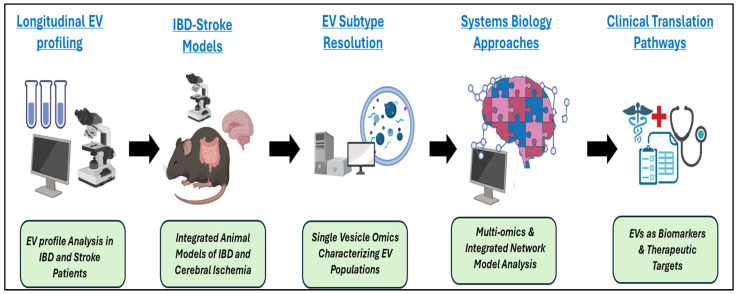
Research priorities within the Gut-Vascular-Brain Axis. This schematic illustrates a translational research pipeline spanning EV profiling, mechanistic modeling, and clinical application. Beginning with longitudinal EV profiling in patients with IBD and stroke, the workflow progresses to integrated animal models that capture interactions between intestinal inflammation and cerebral ischemia. Subsequent EV subtype resolution leverages single-vesicle omics to characterize heterogeneous EV populations. Systems biology approaches then integrate multi-omics datasets into network-level models to identify key molecular pathways. The final stage highlights clinical translation, emphasizing the development of EV-based biomarkers and therapeutic targets for disorders involving the gut-vascular-brain axis.

Collectively, these priorities frame the next phase of investigation, which is moving from association to causation, from EV profiling to mechanistic resolution, and from conceptual plausibility to clinical consequence (Figure 2). Establishing whether EV-mediated signaling is a modifiable determinant of stroke risk in IBD will determine whether the gut-vascular-brain axis represents a paradigm shift in understanding extraintestinal vascular risks and complications in IBD.

## 9. Translational Implications of EVs in IBD-Associated Stroke 

EVs hold considerable promise as clinically actionable biomarkers for assessing IBD associated vascular and cerebrovascular risk. However, their translational utility depends on standardized measurement approaches and rigorous validation. Clinically feasible assays would rely on EV quantification from routine biofluids such as plasma or serum, with strict pre-analytical control in accordance with MISEV guidelines [48,161]. Core measurements would include EV abundance, cell-of-origin markers (e.g., platelet, endothelial, and immune-derived EVs), characterization of the cargo, including, pro-inflammatory cytokines, procoagulant factors, inflammatory miRNAs including miR-155, miR-21, and miR-146a. Complementary exploratory markers, such as EV associated lncRNAs and lipid signatures, may further enhance the discrimination of EV specific cargo [162,163].

The analytical pipeline to validate EV as a biomarker would ideally progress through staged validation, i.e., initial demonstration of technical feasibility, cross-sectional association with disease activity (e.g., active vs. remission; IBD patients with vs. without stroke), prospective prognostic evaluation of stroke among IBD patients, and ultimately external replication across independent cohorts (principles aligned with MISEV2018 and vascular EV literature such as Ref. [164]). Compared with CRP, which reflects generalized systemic inflammation induced by IBD, EV cargo profiles may provide specific insight into the future risk of endothelial activation, coagulation pathways, and thrombo-inflammatory signaling relevant to cerebrovascular risk [165,166]. Importantly, to determine whether EV markers predict stroke risk better than CRP, direct comparisons using methods such as receiver operating characteristic (ROC) curves, reclassification tests, and decision-curve analysis will be needed [167]. Ultimately, a standardized EV derived risk score could complement existing laboratory markers and help guide preventive strategies in high-risk IBD populations [161].

Beyond diagnostics, EVs may be therapeutic targets in IBD-associated stroke [168]. Options include reducing EV release by inhibiting nSMase2/ceramide pathways or Rab27-dependent secretion [169,170]; neutralizing circulating EVs by masking procoagulant phosphatidylserine or tissue factor (e.g., annexin-based approaches [171]); selectively depleting thrombogenic subpopulations such as P-selectin-positive platelet EVs [172]; or interrupting EV uptake/downstream signaling in endothelial cells, including TLR and cGAS–STING inhibition, when microbial EVs contribute to BBB dysfunction [173]. Translation will require phase-specific clinical studies under harmonized pre-analytics, multi-site standard operating procedures (SOPs), and integration of single-EV omics [48].

## 10. Conclusions

Stroke is increasingly recognized as a clinically significant extraintestinal complication of IBD, with epidemiologic studies demonstrating a persistent elevation in ischemic risk that cannot be fully explained by traditional vascular factors. Although chronic systemic inflammation, hypercoagulability, and endothelial dysfunction provide a partial mechanistic framework, emerging evidence suggests that EVs may function as integrative mediators linking intestinal inflammation to cerebrovascular vulnerability. Determining whether EVs function as causal drivers, amplifiers, or secondary biomarkers of systemic inflammation will require longitudinal human cohort studies, functional vesicle-transfer experiments, and integrated multi-omics approaches. If validated, EV signatures could serve not only as biomarkers of cerebrovascular risk in IBD but also as novel therapeutic targets to attenuate thrombo-inflammatory signaling. Advancing this work will require bridging gastroenterology and vascular neuroscience through focused translational investigation to establish whether modulation of EV pathways can meaningfully reduce stroke risk in this population.

## Figures and Tables

**Table 1:** Epidemiologic Evidence Linking IBD to Stroke Risk.

Study	Design & Timeframe	Population	Effect Estimate	Subgroup Findings	Ischemic (IS) vs. Hemorrhagic Stroke (HS)	Key Adjustments	Interpretation
Luo et al., 2025 [1]	Systematic review & meta-analysis	International cohort studies	HR ≈ 1.30 for overall stroke	Elevated risk in both CD and UC	IS: consistently increased (~1.25–1.35). HS: weaker or non-significant increase in most cohorts.	Age, sex	Confirms moderate but consistent increased risk, largely driven by ischemic events
Chen et al., 2021 [2]	PRISMA-compliant meta-analysis	Multi-cohort	OR/RR ≈ 1.21	Risk present in both CD (~1.35) and UC (~1.15)	IS: significantly increased. HS: smaller effect; some studies non-significant.	Age, sex	Strengthens association; effect size modest but robust
Sun et al., 2023 [3]	Population-based, sibling-controlled cohort (1969–2019)	>85,000 biopsy-confirmed IBD patients	Adjusted HR ≈ 1.13–1.14	Stronger association in women and younger patients	IS: modest but persistent elevation. HS: no strong or consistent excess risk.	Extensive multivariable adjustment incl. socioeconomic & comorbidities	Long-term persistent ischemic stroke risk supports disease-specific inflammatory mechanisms.
Fan et al., 2023 [15]	Meta-analysis of cohort studies	International	15–30% relative increase	Younger patients show relatively higher excess risk	IS: pooled significant increase. HS: heterogeneous; generally weaker association.	Age, sex	Highlights disproportionate risk in individuals with lower baseline cardiovascular risk
Xiao et al., 2015 [5]	Cohort meta-analysis	Multiple regions	Increased risk across studies	Both ischemic and hemorrhagic reported	IS: elevated risk observed. HS: signal less consistent and often non-significant.	Variable	Early epidemiologic signal; ischemic events appear to predominate.

**Table 2:** Integrated Classical and EV Mediated Mechanisms Linking IBD to Ischemic Stroke Risk *.

Pathway	Key Mediators	EV-Mediated Amplification	Cerebrovascular Effects	Stroke Relevance	Evidence Strength
Chronic systemic inflammation	TNF-α, IL-6, CRP [27,28,29,30]	EV cargo containing cytokines, inflammatory miRNAs (e.g., miR-155, miR-21) that activate endothelial NF-κB and adhesion pathways [10,106]	Endothelial activation, oxidative stress	Accelerated ischemic stroke risk	High (Classical)/Moderate-Emerging EV
Endothelial dysfunction	Reduced NO, adhesion molecule upregulation [28,31,32,33,34,35]	Endothelial and platelet-derived EVs expressing ICAM-1, tissue factor, pro-inflammatory miRNAs; EVs impair eNOS/NO signaling and increase ICAM-1/VCAM-1 [105,107]	Impaired vasodilation, pro-adhesive phenotype, vascular priming	Cerebral small vessel dysfunction and BBB vulnerability	High (Classical)/Moderate (EV)
Hypercoagulability	↑ Fibrinogen, Factor VIII, platelet activation [19,43]	Tissue factor-positive EVs, phosphatidylserine exposure enhancing thrombin generation and fibrin formation [45,97]	Enhanced coagulation cascade activation and platelet aggregation	Arterial thromboembolism (ischemic stroke predominance)	High (Classical)/Moderate–Strong (EV)
Microbiome dysbiosis	↑ TMAO, ↓ SCFAs signaling [36,37,38,39]	Dysbiosis modulates host EV cargo; bEVs translocate and activate endothelial/microglial TLR/cGAS–STING pathways [46,111]	Endothelial injury, systemic inflammation	Thrombo-inflammatory priming	Moderate (Classical)/Emerging (EV)
BBB Disruption	Cytokine-mediated permeability changes [143,144]	EV-borne miRNAs (e.g., miR-155/miR-21) and inflammatory cargo disrupt tight-junction proteins (claudin-5, occludin, ZO-1) and increase permeability [130,135]	Increased BBB permeability, neurovascular unit activation	Facilitates neuroinflammation and secondary thrombosis	Moderate (Classical)/Emerging but Mechanistically Compelling (EV)
Genetic susceptibility	Immune & coagulation polymorphisms [145,146]	Potential genotype-dependent EV cargo variability (hypothesized)	Variable inflammatory and thrombotic responsiveness	Explains heterogeneity in stroke risk	Emerging

* Classical mechanisms are supported by established IBD and stroke literature, whereas EV-mediated pathways represent emerging amplifiers supported by experimental and translational studies cited in the main text.

## Data Availability

Not applicable. No new data was created or analyzed in this study.

## Abbreviations

The following abbreviations are used in this manuscript:

bEVs	bacterial Extracellular Vesicles
BBB	Blood-brain barrier
CD	Crohn’s Disease
cGAS–STING	Cyclic GMP-AMP synthase- Stimulator of Interferon Genes
CNS	Central nervous system
CRP	C-Reactive Protein
DAMPs	Danger-associated Molecular Patterns
eNOS	Endothelial nitric oxide synthase
EV	Extracellular Vesicle
HR	Hazard Ratio
HS	Hemorrhagic Stroke
HMGB1	High-mobility group box 1
IBD	Inflammatory Bowel Disease
ICAM-1	Intercellular adhesion molecule-1
IEC	Intestinal epithelial cell
IL	Interleukin
IS	Ischemic stroke
ISEV	International Society for Extracellular Vesicles
lncRNA	long non-coding ribonucleic acid
LPS	Lipopolysaccharides
MHC	Major histocompatibility complex
miRNA	Micro Ribonucleic Acid (microRNA)
MISEV	Minimal Information for Studies of Extracellular Vesicles
MyD88	Myeloid differentiation primary response 88
nSMase2	Neutral sphingomyelinase 2
NET	Neutrophil extracellular trap
NF-κB	Nuclear factor kappa-light-chain-enhancer of activated B cells
NLRP3	NOD-, LRR- and pyrin domain-containing protein 3
NO	Nitric oxide
OR/RR	Odds Ratio/Relative Risk
PRISM	Preferred reporting items for systematic reviews and meta-analyses
RAGE	Receptor for Advanced Glycation End products
ROC curve	Receiver Operating Characteristic curve
ROS	Reactive oxygen species
SCFA	Short chain fatty acid
TLR	Toll-like receptor
TMAO	Trimethylamine N-oxide
TNF	Tumor Necrosis Factor
UC	Ulcerative Colitis
VCAM-1	Vascular cell adhesion molecule-1
ZO-1	Zonula occludens-1
